# Sexual and reproductive health of young people with disability in Ethiopia: a study on knowledge, attitude and practice: a cross-sectional study

**DOI:** 10.1186/s12992-016-0142-3

**Published:** 2016-02-10

**Authors:** Tigist Alemu Kassa, Tobias Luck, Assegedech Bekele, Steffi G. Riedel-Heller

**Affiliations:** Institute of Social Medicine, Occupational Health and Public Health, Medical Faculty, University of Leipzig, Philipp-Rosenthal-Straße 55, 04103 Leipzig, Germany; LIFE—Leipzig Research Center for Civilization Diseases, University of Leipzig, Leipzig, Germany; Gondar College of Medicine and Health Sciences, School of Medicine, University of Gondar, Gondar, Ethiopia

**Keywords:** Knowledge, Attitude, Practice, Sexual reproductive health, Young people, Disability

## Abstract

**Background:**

As is common in developing countries, in Ethiopia young people with disabilities (YPWD) are more likely than the general population to be illiterate, unemployed and impoverished. They often lack equal access to information and education for reasons ranging from barriers regarding physical access to services to varied special learning needs. Very little is known about knowledge, attitude and practice (KAP) of YPWD regarding sexual and reproductive health (SRH) related issues. We, therefore, aimed to assess the KAP of 426 YPWD in Addis Ababa, Ethiopia.

**Methods:**

A cross-sectional survey was conducted in 2012. Data were collected by trained interviewers using a structured questionnaire covering socio-demographic information, as well as information on KAP regarding SRH.

**Results:**

Only 64.6 % of YPWD were aware of SRH services. Radio and TV were mentioned as the main sources of information by 62.2 % of the participants. 77.9 % had never had a discussion about SRH topics with their parents. Even though 96.7 % of the respondents had heard about HIV, 88 % had poor knowledge about ways of preventing HIV. Perception of the risk of getting infected with HIV was found to be generally low in YPWD; only 21.6 % believed that they were at risk of acquiring HIV.

**Conclusions:**

Our study, in general, demonstrated that there is a lack of comprehensive knowledge, appropriate practice and favorable attitude of YPWD regarding different SRH-related issues. Our findings thus clearly indicate the need for strategies and programs to raise SRH-related awareness and to help YPWD to develop the appropriate skills and attitudes needed for a healthy reproductive life.

## Background

Young people with disabilities (YPWD) commonly face more discrimination and severe social, economic, and civic disparities than their non-disabled peers. For many YPWD, exclusion, isolation, and abuse, as well as lack of educational and economic opportunities are commonplace. As a group, YPWD are amongst the most marginalized and poorest youth in the world. Their basic rights are not well met and societal acceptance is often out of reach [1, 2]. People with disabilities also have poorer health outcomes, lower education achievements, less economic participation and higher rates of poverty than non-disabled people. One reason for this disparity is that people with disabilities experience barriers to accessing services. These difficulties are exacerbated in less advantaged communities [3]. The large majority of people with disabilities live in developing countries, often under poor conditions, lacking the basic support and services that would improve their lives considerably [4].

Education is critical for all children to realize their full potentials. However, UNESCO estimates that only 1–2 % of children with disabilities in developing countries attend school. By the time they enter adolescence, many YPWD run a high risk of being illiterate, leading to restricted opportunities for further education, employment, and income generation. Some families do not feel that children with disabilities should receive an education, often believing that young people with disabilities are incapable of learning (Groce, 2004). In societies that favor males, young women with disabilities are at a particular disadvantage, as families may be reluctant to allocate resources to them. According to a UNESCO report, boys with disabilities attend school more frequently than girls with disabilities and 99 % of girls with disabilities are illiterate [1, 5, 6].

Access to reproductive health information is often not disseminated or available to YPWD. For example, in several developing countries YPWD often do not receive advice on HIV/AIDS, as the clinics are physically inaccessible, material is not available for those with visual impairments, and providers are unable to communicate in sign language. Moreover, most health care professionals have no disability awareness and, consequently, feel unwilling or unable to address their issues [5].

Society tends to think that people with disability should be non-sexual. In many cases, sexuality education is withheld because it’s assumed the person ‘won’t need it’. Some people hold the misconception that people with disability shouldn’t have fulfilling sex lives or have access to sexuality education, in case it ‘gives them ideas’ [4]. People with disability also find that information on sexual and reproductive health is often inaccessible to them [1, 4, 7].

Adolescents and young people in Ethiopia are generally at an increased risk of sexual and reproductive health (SRH) related problems. Despite these immense problems, they have limited access to quality sexual and reproductive health services. Also, there are only few national programs specifically targeted to addressing the needs of this group [8]. The health seeking behavior of young Ethiopians, particularly in relation to their sexual and reproductive health, is very limited even when compared to many African countries. Some of the behaviors and challenges can be attributed to the lack of youth-friendly services that reassure confidentiality, service providers’ biases against this section of the population, and low level of awareness among members of the community [9].

YPWD in Ethiopia, as in other developing countries, have an increased risk of SRH-related problems, as they are more likely than the general population to be illiterate, unemployed and impoverished. They often lack equal access to information and education for reasons ranging from physical access to classrooms and service areas to varied special learning needs. Values and attitudes of others, and their decisions about what education or information to provide, also play a significant role. There is also a lack of useful, youth and disability friendly, and easy accessible information related to sexuality and relationships for YPWD. In addition there is a lack of positive, open and respectful spaces for YPWD to discuss disability and sexuality/relationship issues. Lack of access to training and educational programs, in turn, hinders the development of qualified personnel. Very little has been done so far concerning dissemination of information and public awareness campaigns to improve public attitudes about persons with disabilities [10]. These issues exacerbate this group’s problems making them vulnerable to different types of SRH-related problems like STI/HIV, unwanted pregnancy, or unsafe abortion.

In a first study published in 2014, we introduced a sample of *n* = 426 YPWD and provided data on their SRH status (e.g., on their sexual activity, age at first sexual activity, risky sexual behavior) as well as the magnitude of SRH related problems (e.g., on STI, unwanted pregnancy, abortion). In this study, we aimed at providing further important information on this unique study sample of YPWD in Ethiopia. Based on the belief that all people have the right to have access to education and information that can help them to make safe and healthy choices and decision about their bodies and relationships, we particularly focused on their knowledge, attitudes and practice (KAP) regarding sexual and reproductive health related issues, as to the best of our knowledge there are no study, that provided such information, so far.

## Methods

### Study design and area

A cross-sectional survey was conducted from June-September 2012 in Addis Ababa, Ethiopia.

### Study population

All disabled youth aged 10 to 24 years residing in Addis Ababa who were enrolled in different support organizations (Ethiopian National Association for Physically Handicapped, Ethiopian National Association for the Blind, Ethiopian National Association for the Deaf, Ethiopian National Association for the Deaf Blind, Ethiopian National Association for Leprosy Patients; Support Organization of the Mentally Handicapped) during the study period.

### Sample size determination and sampling technique

The sample size was determined using the formula for single population proportion. Based on a significance level of 95 % (α = 0.05), a five percent margin of error, and an assumption of 50 % prevalence of SRH-related problems among study subjects, a total of 426 YPWD were included in the study. A probability sampling method was used to achieve the required sample size after census was conducted to get the sampling frame in each organization. The total sample size was then proportionally allocated to all organizations of people with disability according to the number of YPWD in the respective organizations. The study subjects were selected by systematic random sampling.

WHO classifies *young people* as those between the ages of 10–24 years [11]. We followed this WHO classification and aimed at assessing the KAP of disabled young people in this age group 10–24 years. We are aware that this group includes the overlapping groups of *adolescents* (WHO: ages 10–19 years) as well as of *youth* (WHO: ages 15–24 years), but even the very young adolescents could already be affected by sexual and reproductive health (SRH) related issues. In our study, however, 99 % (*n* = 421) of the young participants were aged between 15 and 24 years. Only 1 % (*n* = 5) were younger (13–14 years) because only few young people of this age group have access to the organization of PWD.

### Inclusion and exclusion criteria

Disabled people aged 10–24 years who were enrolled as members of one of the organizations at the time of data collection were included in the study. Those who were critically ill at the time of study and unable to communicate and respond to questionnaires were excluded from the study.

### Research instrument, measurements and data collection

The data were collected by trained interviewers using a pretested structured questionnaire covering socio-demographic information as well as information on KAP regarding SRH-related issues.

The knowledge of SRH-related issues was assessed with 10 multiple-choice questions—each with 6 to 12 correct responses. The corresponding maximum score for each question was 12, 8 or 6; the minimum score was 0 points. Based on a modification of Bloom’s cut-off points from Nahida’s KAP-Study (2007) [12], we categorized the scores of each question into three levels of knowledge:

- Good knowledge: a score of 80–100 % (9–12, 7–8 and 5–6 out of 12, 8 and 6 points),
- Moderate knowledge: a score of 50–79 % (6–8, 4–6, and 3–4 out of 12, 8 and 6 points),
- Poor knowledge: a score <50 % (0–5, 0–3 and 0–2 out of 12, 8 and 6 points).

Attitudes on SRH-related issues were assessed with nine attitudinal statements (e.g. “Early age premarital sex for girl is acceptable”). Based on three point Likert scales for each statement, participants could chose between three possible response categories: “agree”, “neutral”, or “disagree”. For positive attitudinal statements, each participant’s response was then designated as either favorable (agreeing with the positive attitudinal statement based on scientific facts) or unfavorable attitude (disagreeing with the positive attitudinal statement based on scientific facts or neutral). For negative attitudinal statements, only participants who chose the response category disagree were considered as having a favorable attitude. We then calculated the overall percentage of favorable attitudes for each YPWD. A similar procedure was used to assess practice of YPWD regarding SRH-related issues. For four practice statements (e.g. “Ever tested for HIV”), participants could chose between presence of practice/intention to practice (considered as good practice = 1 point) or absence (considered as poor practice = 0 points) [13].

The KAP questionnaire has also been pretested on YPWD aged less than 15 years and was found suitable for assessing participants of this young age.

### Ethical approval

Ethical approval was obtained from the ethics committee of Addis Ababa University. Written informed consent was obtained from the study subjects and guardians before collecting the data. All personal data were treated confidentially by omitting the names of the respondents from the questionnaires. Only pseudonymized data were analyzed.

### Data analysis

Data were analyzed using Predictive Analytics Software (PASW), version 20. All analyses employed an alpha level for statistical significance of 0.05 (two-tailed). Group differences were analyzed using *t*-test, Mann–Whitney-*U*-test, and *χ*^2^-test, as appropriate. We used unadjusted and adjusted logistic regression models to assess putative determinants of YPWD awareness about sexually transmitted infections (STIs) and SRH services. For each variable included in the regression models, odds ratios (ORs) and 95 % confidence intervals (95 % CI) were calculated.

## Results

### Study sample of young people with disability

A total of 426 young people with disabilities aged 10–24 years old who were members of various disability associations participated in this study. The mean age was 20.8 (SD+/−2.6) years. Of the 426 respondents, 64.3 % (*n* = 274) were males and 35.7 % (*n* = 152) females. The majority (81.2 %, *n* = 346) of the respondents were literate and 67.4 % (*n* = 287) identified themselves as Orthodox Christians.

Concerning marital status, most of the respondents (65.5 %, *n* = 279) were single. Regarding the forms of disability, impairments were as follows: those with impaired mobility 41.5 % (*n* = 177), visual impairment 23 % (*n* = 98), hearing impairment 19.2 % (*n* = 82), partial mental impairment 13.1 (*n* = 56) and multiple impairments 3.1 % (*n* = 13). Most YPWD (47.9 %, *n* = 204) became disabled during their early childhood. Regarding the cause of disability, the majority of the respondents (44.1 %, *n* = 188) indicated a specific disease as the cause for their disability, followed by accident (28.2 %, *n* = 120) and disability from birth (congenital; 19.2 %, *n* = 82).

Thirty-six percent (*n* = 154) of the respondents lived with their parents. Only 43.7 % (*n* = 186) of the respondents were engaged in some kind of paid job outside of the home. Among them, 38.2 % (*n* = 71) earned 10–20 birr per day (≈ ≤ 1 USD). Most of the respondents (45.1 %, *n* = 192) perceived their parents economic status as poor relative to their neighbors.

### Knowledge of young people with disability regarding sexual and reproductive health issues

The results of SRH-related awareness of the YPWD are presented in Table 1. More than half of the respondents (64.6 %, *n* = 275) had heard about SRH services, but only 26.1 % (*n* = 111) had ever utilized them. The majority of the respondents (70.2 %, *n* = 299) did not know the period during the menstrual cycle when pregnancy is most likely to occur. Most of the respondents (84.5 %, *n* = 360) had heard about means of avoiding pregnancy, i.e. family planning (FP) methods. To assess the depth of knowledge of the YPWD regarding FP methods, respondents were asked about different methods that can prevent pregnancy. The majority (66.7 %, *n* = 284) had poor knowledge about the different methods (less than five out of 12 methods known); the mean number of known methods was 4.4 (SD 2.5). Condoms, oral contraceptive pills, and injectables were the most known FP methods (see Table 1).

**Table 1:** Knowledge of young people with disability regarding sexual and reproductive health issues 2012, Addis Ababa (*n* = 426)

*Knowledge*	*Young people with disability*	
	*n*	*%*
Awareness of pregnancy risk times in the menstrual cycle		
Yes	127	29.8
No	299	70.2
Ever heard of SRHS		
Yes	275	64.6
No	151	35.4
Ever heard of FP methods		
Yes	360	84.5
No	66	15.5
FP methods (most known method)^a^		
Oral contraceptive pills	246	68.3
Condoms	255	70.8
Injectables	218	60.6
Norplant	143	39.7
IUDs	125	34.7
Sterilization	23	6.4
Abstinence	28	7.8
Withdrawal	35	9.7
Washing the genitalia after intercourse	4	1.1
Intercourse in the up right position	4	1.1
Safe period/abstinence	66	18.3
Other	19	5.3
Ever heard of STIs		
Yes	326	76.5
No	100	23.5
Most known types of STIs^a^		
Gonorrhea	250	76.7
Syphilis	231	70.9
Chanchroid	155	47.5
Lymphogranuloma venerum	103	31.6
HIV/AIDS	223	68.4
Other	4	1.2
Knowledge about important STI signs/symptoms^a^		
Genital ulcer	156	53.2
Genital discharge	177	60.4
Pain during urination	193	65.9
Genital swelling	60	20.5
Don’t know	133	31.2
Others	9	3.1
Ever heard of HIV		
Yes	412	96.7
No	14	3.3
Knowledge about ways of HIV transmission^a^		
Unsafe sexual intercourse	374	90.8
Sharing needles and syringes	329	79.9
Blood transfusion	109	26.5
During pregnancy and child birth	97	23.5
Through breast milk	47	11.4
Through mosquito and other insect bite	11	2.7
Casual contact with a person (Hand shaking & sharing food…)	7	1.7
Others	5	1.2
Knowledge about ways of HIV prevention^a^		
Abstain from sexual intercourse	265	65.9
Use condom in every act of sexual intercourse	252	62.7
Remain faithful to a partner	221	55.0
Avoid contaminated sharp objects	95	23.6
Avoid unsafe injections	60	14.9
Avoid casual sex	44	10.9
Avoid sex with CSWs	22	5.5
Others	6	1.5
Ever heard of VCT		
Yes	364	85.4
No	62	14.6
Main source of SRH information		
TV/Radio	265	62.2
Newspaper	41	9.6
School teachers	115	27.0
Parents	40	9.4
Partners	8	1.9
Friends	120	28.2
Health professionals	100	23.5
Associations	42	9.9
Trainings	6	1.4
None (I have no idea)	5	1.2
Others	7	1.6

*CSW* commercial sex worker, *FP* family planning, *IUD* intra uterine device, *SRHS* sexual reproductive health service, *STIs* sexually transmitted infections, *VCT* voluntary counseling and testing

^a^Multiple answer question

Among the 426 respondents, 76.5 % (*n* = 326) had heard about diseases that can be transmitted through sexual contact (sexually transmitted infections/STIs). To establish depth of knowledge of the YPWD on STIs, respondents were asked about specific types of illnesses that can be transmitted sexually. We found that the majority of the respondents (53.3 %, *n* = 227) had poor knowledge about the different types of STIs (less than two out of six points). The mean knowledge score was 2.3 (SD 1.7); gonorrhea, syphilis and HIV/AIDS were the most known types of STIs (see Table 1). Our study also showed that there were YPWD who did not link HIV with sexually transmitted infection.

The majority of the respondents (96.7 %, *n* = 412), however, have generally heard about HIV. To determine the depth of knowledge of HIV transmission and prevention, respondents were asked specific questions as to how one can get the HIV virus and how one can protect oneself against it. We found that the majority of the respondents (83.8 %, *n* = 357) had moderate knowledge about ways of HIV transmission (four to six out of 8 points). The mean knowledge score was 5.1 (SD 1.4). Unprotected sexual intercourse, sharing of sharp objects, blood transfusion, pregnancy, childbirth, and breast milk were the most known ways of HIV transmission (see Table 1). The majority of the respondents (88.0 %, *n* = 375), however, had poor knowledge about means of HIV prevention (less than three out of 8 points). The mean knowledge score was 2.3 (SD 1.5). Abstinence, using a condom, and faithfulness to one sexual partner were stated most frequently as ways of HIV prevention (see Table 1). Regarding access to SRH information, half of the respondents (49.8 %, *n* = 212) stated that they perceived YPWD as not well informed about SRH. TV/Radio were stated as the main source of SRH information (62.2 %, *n* = 265) followed by information from friends (28.2 %, *n* = 120), school teachers (27 %, *n* = 115) and health professionals (23.5 %, *n* = 100; see Table 1).

### Attitudes and practice of young people with disability regarding sexual and reproductive health issues

The results on SRH-related attitudes and practices of the YPWD are presented in Table 2. Half of the respondents (52.1 %, *n* = 222) perceived services as inaccessible to people with disabilities. Reasons for inaccessibility were inconvenience of the service areas for people with disabilities (62.2 %, *n* = 138) followed by lack of information (43.7 %, *n* = 97), providers’ disapproval (33.3 %, *n* = 74), lack of money (26.6 %, *n* = 59), being afraid to go for the service (23.0 %, *n* = 51), and parents’ disapproval (13.1 %, *n* = 29).

**Table 2:** Attitudes and practice of young people with disability regarding selected sexual and reproductive health issues 2012, Addis Ababa (*n* = 426)

*Attitude statements*	*Young people with disability*			
	*Favorable attitude*		*Unfavorable attitude*	
	*n*	*%*	*n*	*%*
A person can get HIV the first time he or she has sex. (favorable attitude = yes; unfavorable attitude = no/neutral)	272	63.8	154	36.2
By looking carefully, one can know if someone has HIV. (favorable attitude = no; unfavorable attitude = yes/neutral)	357	83.8	69	16.2
Early age premarital sex for boys is supported. (favorable attitude = no; unfavorable attitude = yes/neutral)	294	69.0	132	31.0
Early age premarital sex for girl is supported. (favorable attitude = no; unfavorable attitude = yes/neutral)	299	70.2	127	29.8
Discussing condom or contraceptive with young people promotes promiscuity. (favorable attitude = no; unfavorable attitude = yes/neutral)	298	70.0	128	30.0
Using condom is a sign of not trusting partner. (favorable attitude = no; unfavorable attitude = yes/neutral)	247	58.0	179	42.0
A wife has a right to refuse unprotected sex with her husband if she wants to use condom and if a husband not. (favorable attitude = yes; unfavorable attitude = no/neutral)	253	59.4	173	40.6
A person having multiple sex partners has a high risk of acquiring HIV. (favorable attitude = yes; unfavorable attitude = no/neutral)	333	78.2	93	21.8
There is no evidence for existence of HIV/AIDS. (favorable attitude = no; unfavorable attitude = yes/neutral)	345	81.0	81	19.0
*Practice*	*Yes*		*No*	
	*n*	*%*	*n*	*%*
Ever utilized any SRH services	111	26.1	315	73.9
Ever tested for HIV	239	56.1	187	43.9
Intention of having an HIV Test	326	76.5	100	23.5
Practice of parent-teen communication	94	22.1	332	77.9
Preferred group for SRH issue discussion^a^				
Friends	235	55.2	191	44.8
Parents	67	15.7	359	84.3
Siblings	28	6.6	398	93.4
Partner	74	17.4	352	82.6
Health Professionals	126	29.6	300	70.4
Others	16	3.8	410	96.2

*AIDS* acquired immune deficiency syndrome, *HIV* human immune deficiency virus, *SRHS* sexual reproductive health service

^a^Multiple answer question

Perception of YPWD about risk of getting infected by HIV was found to be generally low; only 21.6 % (*n* = 92) believed that they are at risk of acquiring HIV. The reasons stated by the YPWD for their increased risk were having had sex without condom (51.1 %, *n* = 47), having had injuries with contaminated sharps (27.2 %, *n* = 25), having had multiple sexual partners (25.0 %, *n* = 23) and have had sexual intercourse with commercial sex worker (CSW; 15.2 %, *n* = 14).

As shown in Table 2, 81 % (*n* = 345) of the respondents correctly rejected the attitude statement that “there is no evidence for existence of HIV/AIDS”. Moreover, 63.8 % (*n* = 272) of the respondents correctly agreed with the statement that “a person can get HIV the first time he or she had sex” and 83.8 % (*n* = 357) correctly disagreed with the statement that “by looking carefully, one can know if someone has HIV”. Most of the respondents (78.2 %, *n* = 333) also had a favorable attitude towards the risk for acquiring HIV by agreeing with the statement, “a person having multiple sex partner has a high risk of acquiring HIV”. However, 40.6 % (*n* = 173) of the respondents, showed an unfavorable attitude towards reproductive health rights by disagreeing with the statement, “a wife has a right to refuse unprotected sex with her husband if she wants to use condom and if her husband does not”. Also, 30.0 % (*n* = 128) and 42 % (*n* = 179) of the respondents had unfavorable attitudes towards the statements, “discussing condom or contraceptive with young people promotes promiscuity” and “using condom is a sign of not trusting partner” respectively.

The majority of the respondents (85.4 %, *n* = 364) had heard about voluntary counseling and testing (VCT), but only 56.1 % (*n* = 239) had been voluntarily counseled and tested for HIV. Among the study population, 76.5 % (*n* = 326) indicated that they would undergo VCT if the service would be available, whereas 23.5 % (*n* = 100 had no such intention. The main reason stated for having had the intention to get tested was to know the sero status (90.8 %, *n* = 296), whereas fear of a positive test result and anticipated discrimination and stigma associated with HIV/AIDS were the main reasons for not having the intention of getting tested (27.3 %, *n* = 21; 24.7 %, *n* = 19). Regarding the practice of parent-teen communication, the majority of the YPWD (77.9 %, *n* = 332) stated that they did not have a discussion on SRH topics with their parents. Most of the respondents preferred to discuss topics of sexuality and reproductive health with friends (55.2 %, *n* = 235), with health professionals (29.6 %, *n* = 126) or with the partner (17.4 %, *n* = 74; see Table 2).

### Putative determinants of sexually transmitted infection awareness by young people with disability in Ethiopia

Table 3 shows our findings on the putative determinants of sexually transmitted infection awareness of YPWD. As shown in the table, respondents’ age, education, and form of disability were significantly associated with awareness of STIs (adjusted model). Every additional year of age yielded an adjusted OR (aOR) = 1.3 (95 % CI = 1.1–1.4) and being literate an aOR = 7.0 (95 % CI = 3.4–14.6; reference illiterate). STI awareness was also higher among respondents with hearing impairment, visual impairment and physical impairment than among those with partial mental impairment (aOR = 10.1, 95 % CI = 3.5–29.4; aOR = 88.3, 95 % CI = 13.7–568.6; aOR = 3.6, 95 % CI = 1.3–10.2).

**Table 3:** Putative determinants of sexually transmitted infections awareness by young people with disability 2012, Addis Ababa (*n* = 426)

*Characteristics*	*Ever heard of sexually transmitted infections*				*Adjusted model*			
	*Yes*		*No*		*Wald*	*d.f.*	*P value*	*Adjusted OR (95 % CI)*
	*n*	*%*	*n*	*%*				
Sex								
Female	112	34.4	40	40.0				Ref.
Male	214	65.6	60	60.0	0.011	1	0.917	1.0 (0.5–1.8)
Age, every additional year	Mean	SD	Mean	SD				
	20.97	2.521	20.11	2.792	10.856	1	0.001	1.3 (1.1–1.4)
Marital status								
Unmarried	201	61.7	78	78.0				Ref.
Married	125	38.3	22	22.0	0.130	1	0.718	1.2 (0.5–2.7)
Religion								
No religion	7	2.1	4	4.0				Ref.
Christian	277	85.0	83	83.0	1.019	1	0.313	2.3 (0.5–12.2)
Muslim	42	12.9	13	13.0	1.643	1	0.200	3.3 (0.5–20.4)
Education								
Illiterate	28	8.6	52	52.0				Ref.
Literate	298	91.4	48	48.0	27.489	1	<0.001	7.0 (3.4–14.6)
Form of disability								
Partial mental impairment	20	6.1	36	36.0				Ref.
Hearing impairment	69	21.2	13	13.0	18.017	1	<0.001	10.1 (3.5–29.4)
Visual impairment	96	29.4	2	2.0	22.241	1	<0.001	88.3 (13.7–568.6)
Impaired mobility	132	40.5	45	45.0	5.873	1	0.015	3.6 (1.3–10.2)
Multiple impairment	9	2.8	4	4.0	3.122	1	0.077	4.5 (0.9–23.8)
Time of disability								
From birth	49	15.0	37	37.0				Ref.
Early childhood	162	49.7	42	42.0	0.331	1	0.565	1.3 (0.6–3.0)
Later in life	115	35.3	21	21.0	3.366	1	0.067	2.4 (0.9–6.1)
Work								
No	179	54.9	61	61.0				Ref.
Yes	147	45.1	39	39.0	0.001	1	0.971	1.0 (0.5–2.2)
Living situation								
With parents	104	31.9	50	50.0				Ref.
With relatives	30	9.2	10	10.0	0.443	1	0.506	1.4 (0.5–4.0)
With friends/peers	60	18.4	11	11.0	0.761	1	0.383	0.6 (0.2–1.9)
With partner	37	11.3	11	11.0	0.857	1	0.354	0.5 (0.2–2.0)
Alone	52	16.0	13	13.0	0.040	1	0.841	0.9 (0.3–2.7)
Orphanage	43	13.2	5	5.0	3.020	1	0.082	3.0 (0.9–10.3)
Total	326	100.0	100	100.0				

*CI* confidence interval, *d.f.* degree of freedom, *OR* odds ratio, *SD* standard deviation

### Putative determinants of sexual reproductive health service (SRHS) awareness of young people with disability

Table 4 shows our findings on the putative determinants of SRHS awareness of YPWD. As shown in the table, respondents’ sex, age, education, forms of disability, and living situation were significantly associated with SRHS awareness (adjusted model). Every additional year of age yielded an aOR = 1.2, (95 % CI = 1.1–1.4), being female an aOR = 2.2, (95 % CI = 1.2–3.8; reference male) and being literate an aOR = 6.5, (95 % CI = 3.1–13.6; reference illiterate) for SRHS awareness. SRHS awareness was also higher among respondents with hearing impairment and visual impairment than among those with partial mental impairment (aOR = 3.2, 95 % CI = 1.3–8.0; aOR = 13.3, 95 % CI = 3.8–46.9). Moreover, those respondents who lived in an orphanage had a higher likelihood of having SRHS awareness (aOR = 4.6, 95 % CI = 1.7–12.5) than those who lived together with their parents.

**Table 4:** Putative determinants of sexual reproductive health service awareness by young people with disability 2012, Addis Ababa (*n* = 426)

*Characteristics*	*Ever heard of sexual reproductive health services*				*Adjusted model*			
	*Yes*		*No*		*Wald*	*d.f.*	*P value*	*Adjusted OR (95 % CI)*
	*n*	*%*	*n*	*%*				
Sex								
Male	175	63.6	99	65.6				Ref.
Female	100	36.4	52	34.4	7.223	1	0.007	2.2 (1.2–3.8)
Age, every additional year	Mean	SD	Mean	SD				
	21.11	2.494	20.15	2.707	9.905	1	0.002	1.2 (1.1–1.4)
Marital status								
Unmarried	159	57.8	120	79.5				Ref.
Married	116	42.2	31	20.5	1.519	1	0.218	1.5 (0.8–2.9)
Religion								
No religion	8	2.9	3	2.0				Ref.
Christian	238	86.5	122	80.8	2.049	1	0.152	0.3 (0.0–1.6)
Muslim	29	10.5	26	17.2	3.500	1	0.061	0.2 (0.0–1.1)
Education								
Illiterate	20	7.3	60	39.7				Ref.
Literate	255	92.7	91	60.3	24.979	1	0.000	6.5 (3.1–13.6)
Form of disability								
Partial mental impairment	17	6.2	39	25.8				Ref.
Hearing impairment	52	18.9	30	19.9	6.204	1	0.013	3.2 (1.3–8.0)
Visual impairment	90	32.7	8	5.3	16.284	1	0.000	13.3 (3.8–46.9)
Impaired mobility	111	40.4	66	43.7	0.657	1	0.418	1.5 (0.6–3.8)
Multiple impairment	5	1.8	8	5.3	0.620	1	0.431	0.5 (0.1–2.5)
Time of disability								
From birth	36	13.1	50	33.1				Ref.
Early childhood	138	50.2	66	43.7	0.371	1	0.542	1.3 (0.6–2.7)
Later in life	101	36.7	35	23.2	3.576	1	0.059	2.2 (0.9–4.9)
Work								
No	147	53.5	93	61.6				Ref.
Yes	128	46.5	58	38.4	0.397	1	0.529	1.2 (0.7–2.3)
Living situation								
With parents	76	27.6	78	51.7				Ref.
With relatives	25	9.1	15	9.9	2.345	1	0.126	2.1 (0.8–5.2)
With friends/peers	53	19.3	18	11.9	0.198	1	0.656	1.2 (0.5–3.0)
With partner	40	14.5	8	5.3	3.270	1	0.071	3.0 (0.9–10.1)
Alone	42	15.3	23	15.2	0.000	1	0.984	1.0 (0.4–2.5)
Orphanage	39	14.2	9	6.0	8.591	1	0.003	4.6 (1.7–12.5)
Total	275	100.0	151	100.0				

*CI* confidence interval, *d.f.* degree of freedom, *OR* odds ratio, *SD* standard deviation

## Discussion

In this study, our aim was to assess the knowledge, attitude and practice (KAP) of young people with disability (YPWD) in Addis Ababa, Ethiopia regarding sexual and reproductive health related issues.

We found that most YPWD (84.5 %) have heard about family planning (FP) even though the majority of the respondents (66.7 %) had poor knowledge about the different methods. Accordingly, the majority of YPWD (76.5 %) also had heard about sexually transmitted infections (STIs), but often had poor knowledge about the different types of STIs (53.3 %). Regarding HIV/AIDS, the most important STI, most respondents (96.7 %) had heard about HIV and showed at least moderate knowledge of ways of HIV transmission (83.8 %). However, 88 % of the respondents had poor knowledge about means of HIV prevention. Being older and being literate were found to be important putative determinants of STI awareness. The study also revealed that those with partial mental impairment were the most uninformed.

Our FP awareness finding of 84.5 % is in line with findings from previous KAP studies conducted in Kampala, Uganda and Jimma, Ethiopia [14, 15], but slightly lower when compared to a finding from a community-based Ethiopian study which reported FP knowledge in 96.2 % of the participants [16]. The latter study, however, only included women (*n* = 361) and encompassed a broader age range (15–49 years) than our study. A comparison of the study results, therefore, has to be made with caution.

Our STI awareness finding of 76.5 % is higher compared to findings of a study from Cameroon [17] which reported a STI-awareness rate of 50 % in the 118 included people with disabilities (PWD), but lower compared to findings from other previous studies reporting STI-awareness rates of 91 % (*n* = 371 PWD from Kampala, Uganda) [14] and 94 % (*n* = 616 PWD from Kenya) [18]. Attempts should, therefore, be made to increase STI-awareness in YPWD in Ethiopia.

A pleasant result of our study was the high HIV-awareness rate of 97 % among the interviewed YPWD—a finding that is in line with findings from several other African studies that also revealed that people with disabilities are aware of HIV. According to our findings, these studies, however, also indicated that young disabled people often have weak knowledge about HIV prevention and misconceptions about its transmission [5, 18–23]. A finding that can also be found in another Ethiopian study, that reported a lack of comprehensive knowledge on HIV transmission in 88.5 % and a lack of comprehensive knowledge on prevention in 54 % of the included 96 participants with disabilities [24].

Perception of the risk of getting infected with HIV was found to be generally low among the YPWD in our study, as only 21.6 % believed that they were at risk of acquiring HIV—a lower result compared to findings from other African studies [14, 18, 25–28]. Our finding of a low risk perception of YPWD in Ethiopia, however, is similar to findings from another Ethiopian study, as only 22.9 % of the included 96 respondents believed that they were at a moderate/high chance of acquiring the AIDS virus [24].

Our voluntary counseling and testing (VCT) awareness findings (85.4 %) is higher compared to findings from a study conducted in Malawi that indicated a VCT awareness rate of 70 % in the 317 included respondents [23]. In a study from Tanzania, 85 % of the included 40 PWD indicated a desire to be tested and 55 % of them revealed that they had been tested for HIV [28]. This is a similar finding compared to our study findings, in which 56.1 % were voluntarily counseled and tested for HIV and 76.5 % of the respondents indicated a desire to be tested, if the service is available.

Practice of parent-teen discussion on sexuality related matters was found to be very low among YPWD in our study, as only 22.1 % of the respondents had a discussion with their parents on SRH topics—a lower result compared to findings from study conducted in Debre Markos, Ethiopia that showed that 36.9 % of the included 697 students had had a discussion with a parent on SRH issues. Regarding the preferred group for discussion, most of respondents in our study (55.2 %), preferred to discuss topics of SRH with friends—similar to findings from the above Ethiopian study in which 56.8 % of the participants chose to discuss SRH issues with their friends [29]. Our finding of low parent-teen communication of YPWD in Ethiopia, however, is also consistent with findings of a study conducted in Uganda and Rwanda that revealed that there is very little real discussion about HIV/AIDS between parents and young people with disability [5]. Attempts should therefore be made to increase awareness of parents, families and the community on the need and importance of communication and discussion of SRH-related topics with YPWD.

Availability and accessibility to information on reproductive health related issues are crucial to YPWD to help them to make informed decisions and to have safe and desirable sexual behavior. In our study, more than half (64.6 %) of the respondents have heard about SRHS, but only 26.1 % had utilized SRHS. Radio/TV and friends were cited as major sources of information for 62.2 % and 28.2 % of respondents. However about half (52.1 %) of respondents perceived that services are inaccessible to PWDs and half 49.8 % believed that YPWD are not well informed about SRH. Our study also revealed that those with partial mental impairment are the most uninformed about the service. This study finding is similar to findings from a study that was conducted in other parts of Africa [5, 14, 28]. For example, a study conducted in Nigeria reveled that for people with sensory impairments certain channels of communication are inaccessible [30]. And with regards to intellectual disability, society frequently takes the view that intellectually disabled people have no right to pursue social and sexual relationships. They have often been completely denied sex education [4]. Attempts should, therefore, be made to identify and address the SRH information needs of YPWD, in particular, young with intellectual disability in Ethiopia.

The result of a study conducted in East Gojjam, Ethiopia (*n* = 1001) revealed that the most common sources of information on STIs/HIV/AIDS were the media (82 %) and neighbors (67 %) for urban and rural out of school adolescents respectively and 55.2 % of the included 1001 participants reported that they had visited health institutions for reproductive health reasons, a higher result than our study findings yielded (26.1 %) [31]. The variation of service utilization between this and our study could be due to the difference in the character of the study subjects (out of school adolescents without disability versus YPWD). Our finding of low service utilization of YPWD could also be indicative of inconvenience of services and lacking sources of information for PWDs in the country.

YPWD in our study had unfavorable attitudes towards reproductive health rights of women with disability; for example, believing that, “a wife has no right to refuse unprotected sex with her husband, if she wants to use condom and if her husband does not”. A significant number of YPWD also had unfavorable attitudes towards condom use. This finding is in line with findings from a study conducted in Kenya showing that a main reason for not using condoms is the perception that using condoms in a relationship implies that one does not trust their partner [18].

### Limitations of the study

Our study only considered YPWD in the capital city of Ethiopia and is not therefore representative for the entire population of YPWD in Ethiopia; particularly for those YPWD living in the rural areas of the country [32]. Also, the study participants were selected by systematic random sampling. For unwilling or absent randomly selected study participants on the date of data collection, the next study participant was substituted from the same organization until we got the required sample. However, we were not able to collect information on the non-responders and a possible selection bias cannot, therefore, be excluded.

## Conclusion

Our study demonstrated that there is a lack of comprehensive knowledge among YPWD regarding SRH-related issues such as awareness of the full range of family planning methods, types of sexually transmitted infections and means of HIV prevention. Our study also showed a poor utilization of SRH services as well as a high percentage of unfavorable attitudes towards SRH service use. The study also revealed that those with partial mental impairment are the most disadvantaged and uninformed.

Findings, thus, clearly indicate the need for strategies to increase SRH awareness in YPWD. Specifically, SRH education programs and services that are tailored to the needs of YPWD are essential; information, education and communication (IEC) materials prepared for the general population must be prepared taking this group of populations into consideration. Such SRH education programs should be integrated in school curriculums of YPWDs starting from primary school onwards thereby enabling the gradual acquisition of information and knowledge necessary to develop the appropriate skills and favorable attitudes needed for a healthy reproductive life.

Attempts should also be made to increase awareness in families and the community for SRH-related issues and disability. Furthermore, organizations of people with disabilities should make meticulous efforts to ensure the SRH-related rights of PWDs. And finally, there is also a need for further studies in the area of SRH-related information and service utilization of YPWD to better understand important factors that prevent YPWD from having comprehensive knowledge, favorable attitude and appropriate practice and to develop and implement tailored approaches that improve the current situation.

## Abbreviations

AIDS: acquired immunodeficiency syndrome
FP: family planning
HIV: human immunodeficiency virus
IEC: information, education and communication
KAP: knowledge, attitude and practice
PWD: people with disability
SRH: sexual reproductive health
SRHS: sexual reproductive health service
STI: sexually transmitted infection
VCT: voluntary counseling and testing
YPWD: young people with disability
